# The Existence of the StartReact Effect Implies Reticulospinal, Not Corticospinal, Inputs Dominate Drive to Motoneurons during Voluntary Movement

**DOI:** 10.1523/JNEUROSCI.2473-21.2022

**Published:** 2022-10-05

**Authors:** Jesus A. Tapia, Takamichi Tohyama, Annie Poll, Stuart N. Baker

**Affiliations:** ^1^Facultad de Ciencias Biologicas, Benemérita Universidad Autónoma de Puebla, C.P. 72000 Puebla, Mexico; ^2^Department of Rehabilitation Medicine I, School of Medicine, Fujita Health University, Aichi 470-1192, Japan; ^3^Faculty of Medical Sciences, Newcastle University, Newcastle upon Tyne NE2 4HH, United Kingdom

**Keywords:** brainstem, corticospinal, motor cortex, reaction time, reticulospinal, startle

## Abstract

Reaction time is accelerated if a loud (startling) sound accompanies the cue—the “StartReact” effect. Animal studies revealed a reticulospinal substrate for the startle reflex; StartReact may similarly involve the reticulospinal tract, but this is currently uncertain. Here we trained two female macaque monkeys to perform elbow flexion/extension movements following a visual cue. The cue was sometimes accompanied by a loud sound, generating a StartReact effect in electromyogram response latency, as seen in humans. Extracellular recordings were made from antidromically identified corticospinal neurons in primary motor cortex (M1), from the reticular formation (RF), and from the spinal cord (SC; C5–C8 segments). After loud sound, task-related activity was suppressed in M1 (latency, 70–200 ms after cue), but was initially enhanced (70–80 ms) and then suppressed (140–210 ms) in RF. SC activity was unchanged. In a computational model, we simulated a motoneuron pool receiving input from different proportions of the average M1 and RF activity recorded experimentally. Motoneuron firing generated simulated electromyogram, allowing reaction time measurements. Only if ≥60% of motoneuron drive came from RF (≤40% from M1) did loud sound shorten reaction time. The extent of shortening increased as more drive came from RF. If RF provided <60% of drive, loud sound lengthened the reaction time—the opposite of experimental findings. The majority of the drive for voluntary movements is thus likely to originate from the brainstem, not the cortex; changes in the magnitude of the StartReact effect can measure a shift in the relative importance of descending systems.

**SIGNIFICANCE STATEMENT** Our results reveal that a loud sound has opposite effects on neural spiking in corticospinal cells from primary motor cortex, and in the reticular formation. We show that this fortuitously allows changes in reaction time produced by a loud sound to be used to assess the relative importance of reticulospinal versus corticospinal control of movement, validating previous noninvasive measurements in humans. Our findings suggest that the majority of the descending drive to motoneurons producing voluntary movement in primates comes from the reticulospinal tract, not the corticospinal tract.

## Introduction

Commands to control voluntary movements are sent from the brain to spinal cord (SC) over a variety of descending pathways. In Old World primates, the corticospinal tract (CST) has developed monosynaptic connections to motoneurons, allowing direct cortical control of the final stage of motor output. Other phylogenetically older pathways like the reticulospinal tract (RST) originate in the brainstem (Lemon, 2008). The RST seems better suited to specifying the broad pattern of muscle activity, whereas the CST can control fine-scale patterns of muscle fractionation, which provide primates with their unique dexterous abilities (Zaaimi et al., 2018a). Direct inputs to motoneurons measured under anesthesia are substantially smaller from the RST than from the CST (Riddle et al., 2009), potentially justifying the usual emphasis on the CST in most considerations of primate motor control. However, both the CST and RST make most synaptic contacts on spinal cord interneurons, rather than motoneurons. Since these connections are hard to assess under anesthesia (Alstermark et al., 1999), reliable measures of the relative importance of CST versus RST drive to motoneurons during voluntary movements are not readily available.

In humans, transcranial magnetic brain stimulation (TMS) provides a straightforward way of activating the CST. By contrast, noninvasive approaches to assess RST function are less readily available. One possible measure asks subjects to react to a visual cue; on some trials, the visual stimulus is combined with a loud sound, which, if presented alone, would be sufficient to elicit an acoustic startle reflex. Reaction times following the loud sound are accelerated compared with the visual stimulus alone; the size of this shortening is referred to as the “StartReact” effect (Valls-Solé et al., 1995). Because the overt startle reflex is known to involve the reticulospinal tract (Davis et al., 1982), StartReact is often assumed to measure RST function. One hypothesis is that the motor program is “downloaded” to the brainstem during motor preparation, and then triggered rapidly and automatically by a loud sound (Valls-Solé et al., 1999; Rothwell, 2006). A role for the RST is supported by studies that show enhanced StartReact in some stroke survivors (Choudhury et al., 2019), consistent with RST strengthening to take over lost CST function and contribute to recovery (Zaaimi et al., 2012, 2018b). After spinal cord injury, increased StartReact correlates with spasticity (Sangari and Perez, 2019), which has been previously suggested to result from excess RST activity (Brown, 1994). However, to date the evidence linking RST to StartReact has been indirect; an alternative hypothesis suggests that reaction time is shortened by the impact of loud sounds on cortical circuits projecting to the CST (Carlsen et al., 2012).

In this study, we investigated the neural mechanisms underlying StartReact to provide insights into the interplay between CST and RST during voluntary movement. Single-neuron recordings from CST cells in the primary motor cortex (M1), from the reticular formation (RF) and the spinal cord were gathered during a voluntary reaction task in macaque monkeys. These experimental data served as input to a computational model, which simulated muscle responses assuming different contributions to motoneuron drive from the CST and RST. As in humans, we found that loud sounds shortened reaction time in monkeys. This could only be reproduced by the model if the majority of inputs to motoneurons arose from the RST. Furthermore, the extent of reaction time shortening varied with the magnitude of RST contribution. This appears to validate use of the StartReact effect as a noninvasive assessment of RST function.

## Materials and Methods

Experiments were conducted in two adult female Macaca mulatta monkeys (monkey V: weight, 5.9 kg; age, 4.4 years; monkey U: weight, 7.1 kg; age, 5.1 years). All procedures were approved by the Newcastle University Animal Welfare and Ethical Review Board and were conducted under personal and project licenses issued by the UK Home Office.

### Behavioral task.

The monkeys were trained to place their right forearm in a close-fitting cast; this attached rigidly to a manipulandum, which rotated in the horizontal plane. The cast held the forearm in pronation. The axis of rotation was coaxial with the elbow joint, allowing measurement of elbow flexion/extension movements. The plane of rotation of the forearm was below the shoulder, so that the shoulder was adducted by ∼45°. A potentiometer attached to the manipulandum shaft measured elbow joint angle, and a torque motor (part #353297, Maxon Motors) allowed forces to be applied under computer control. Five large buttons (diameter, 35 mm) were placed with their centers 200 mm from the shaft, equally spaced at 20° angles, with the central button corresponding to an elbow angle of ∼90°. The central button could be illuminated by a white light-emitting diode (LED), and served as a “home” position; the four peripheral buttons could be illuminated either green or red using bicolor LEDs and acted as movement targets.

**Figure 1.**
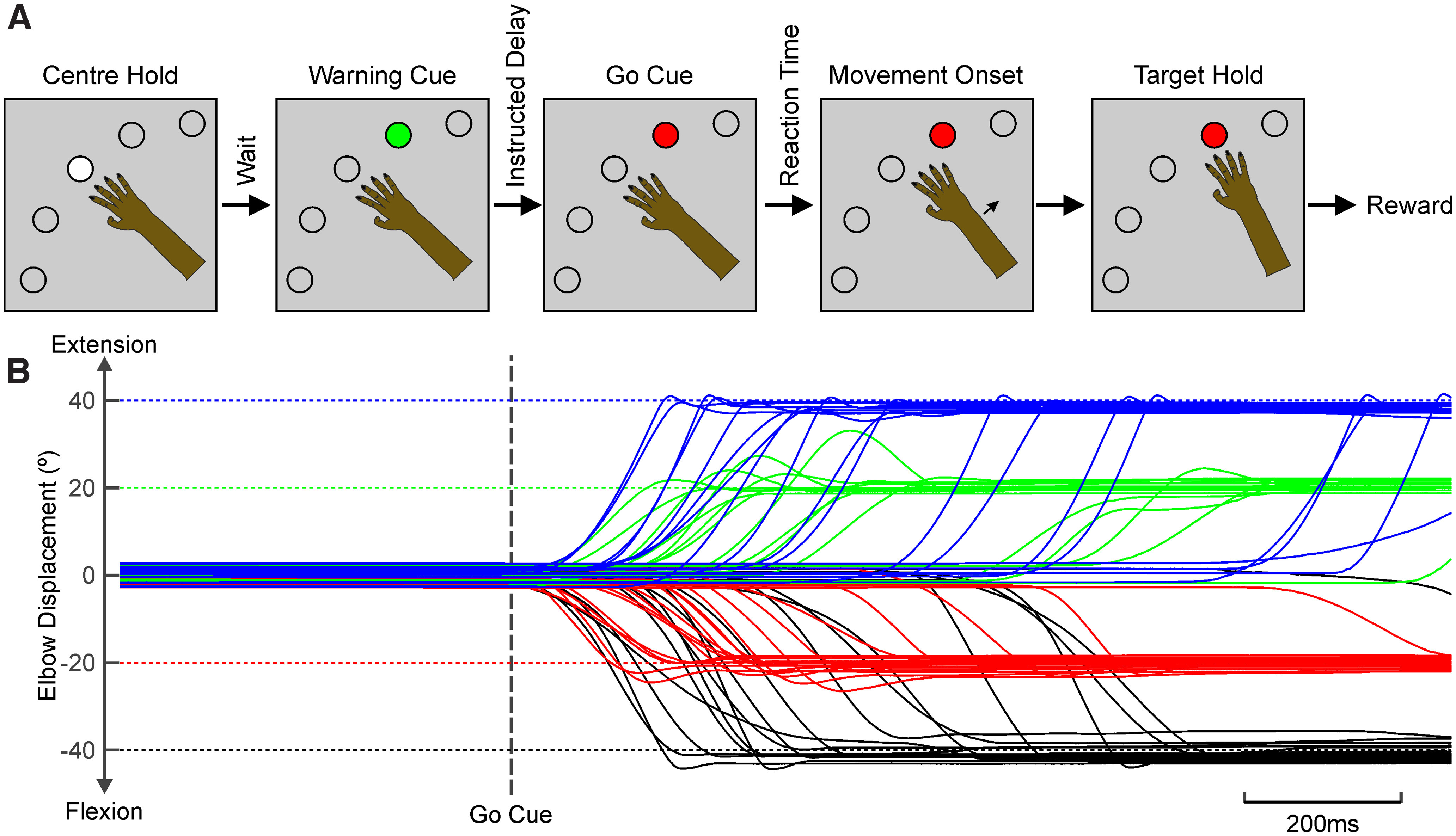
Behavioral task. ***A***, Schematic illustration of the sequence of the behavioral task. The forearm was moved passively by a torque motor to the central position, and the central button illuminated white. One of the four targets then illuminated green, acting as an instructional cue. The target turned red as a go cue, and the monkey moved the arm to align to the target and obtain a reward. ***B***, Example lever movement recordings during performance of trials to different targets, shown by the different colors. Horizontal dotted lines indicate the target positions. Traces are aligned to the go cue.

The sequence followed for the task was as shown in Figure 1*A*. A trial began by the forearm being moved passively by the motor to the home position, using a proportional-integral-derivative (PID) feedback controller. Three hundred milliseconds after the home position was reached, the motor force was switched off, and the central white LED was illuminated. After a delay of 1 s, the white LED was extinguished, and one of the four target buttons was illuminated green. This served as an instructional cue; the animal was required to remain still, and movement away from the central home position resulted in a failed trial. After an instructed delay period of 1 s, the target turned red; this “go” signal cued the animal to make a rapid movement to that location. The arm was required to be placed within ±3° of the target and to hold in this window for 0.4 s. The trial was then counted as a success, and a food reward was given. Auditory cues signaled the instructional cue (500 Hz tone) and the completion of a successful trial (600 Hz tone). Trials in which the target was not acquired within 1.5 s after the go cue were treated as failed trials (300 Hz tone). Auditory cues were delivered at a low sound intensity (∼80 dB). After either a successful or a failed trial, the sequence repeated with the return of the forearm to the central home position under PID control. Figure 1*B* shows overlain traces of elbow flexion/extension angle for trials with different instructed targets, aligned to the go cue.

Animals were trained on this task in stages. Initially, the arm was free, and animals moved to press the illuminated buttons (which contained microswitches) as their instructed response. They were then trained to accept the cast but continued to respond by pressing the buttons. Finally, they were trained to accept the passive movement to the home position in between trials, and a successful response was measured from the forearm position entering the required window, rather than by a button press.

### Loud sound stimulus.

Four possible stimuli were given at the same time as the red target illumination (go cue), with equal probability (0.25). These were no additional stimulation (control), a loud sound, a perturbation in the flexion direction, and a perturbation in the extension direction. The perturbations were delivered by the torque motor and were used for a separate study into set-related reflex modulation; they will not be considered further in this article. The loud sound comprised a 50 ms, 1000 Hz sinusoidal tone, played through stereo speakers to achieve a sound intensity of 115 dB (C weighted).

### Surgical implants.

Sometime during behavioral training, an MRI scan of the head was taken under general anesthesia. This was used to generate a digital model of the skull shape, and to design an annular headpiece with underside shaped to conform to the skull contours. The headpiece was then manufactured in TekaPEEK plastic using a computer-controlled milling machine (monkey V) or in titanium using 3D printing (monkey U).

After behavioral training was complete, the monkeys underwent one or two sterile surgical procedures under general anesthesia. For all surgeries, after initial sedation with ketamine (10 mg/kg, i.m.), anesthesia was induced with propofol (0.6–6 mg/kg, i.v.), and then maintained with inhalation of sevoflurane (1.5–3%) or desflurane (6–7%) in 100% O_2_, with continuous intravenous infusion of alfentanil (12–30 μg/kg/h). The airway was protected with a tracheal tube. Intravenous fluids were given to prevent dehydration (Hartmann's solution; total rate including drug infusions, 10 ml/kg/h). An infusion of methylprednisolone (5.4 mg/kg/h, i.v.) was given to reduce cerebral edema. The animal was kept warm with a thermostatically controlled heating blanket and also a supply of warm air. Positive-pressure ventilation was used. Monitoring during surgery included pulse oximetry (blood oxygen saturation and heart rate), noninvasive blood pressure, core and peripheral temperature, and end-tidal CO_2_. After surgery, postoperative analgesics (meloxicam 0.3 mg/kg, i.m.; buprenorphine 20 µg/kg, i.m.; paracetamol 10 mg/kg, i.v.) and prophylactic antibiotics (either cefotaxime, 20 mg/kg, i.v.; or enrofloxacin, 10 mg/kg, i.v.) were given, with repeated doses as required.

During the first surgery in monkey V, fine wire electrodes were implanted into the brachioradialis, biceps, triceps, flexor digitalis superficialis, and flexor carpi radialis muscles of the right arm; these wires were preattached to a connector, which was tunneled subcutaneously to the back and stored under the skin in a pouch made of silicone rubber. In a second surgery, the headpiece was implanted to allow atraumatic head fixation, along with stainless steel chambers to provide access to the left motor cortex and the brainstem reticular formation. The headpiece was attached to the skull using the system of expanding bolt assemblies and discs described by Lemon (1984). The EMG connector was retrieved from the back and tunneled up to the headpiece. In monkey U, these two surgeries were combined into one; additionally, the chambers were integrated into the titanium headpiece design for monkey U.

After recovery from surgery was complete, a brief procedure under sedation (initial dose of ketamine 10 mg/kg and medetomidine 8 μg/kg, i.m., followed by continuous intravenous infusion of ketamine, 3.3 mg/kg/h, with supplemental intravenous doses as required) implanted two tungsten stimulating electrodes (catalog #LF501G, Microprobes) in the pyramidal tract for antidromic identification of corticospinal neurons (Baker et al., 1999). This used the double-angle stereotaxic approach described in the study by Soteropoulos and Baker (2006), with initial stereotaxic targets anterior 2 mm, left 1 mm, ventral 6 mm; and posterior 3 mm, left 1 mm, ventral 9 mm, expressed relative to the interaural line. Electrodes were fixed at the point with the lowest threshold to elicit an antidromic field potential in epidural recordings overlying M1.

After recordings from M1 and the reticular formation were complete, a further surgery implanted a chamber over the spinal cord (Perlmutter et al., 1998). Vertebrae from C4 to T2 were fused using screws in the lateral mass and dental acrylic, and a laminectomy was performed on vertebrae C5–C7, exposing spinal segments C5–C8.

### Single-unit recordings.

Recordings from M1 used glass-insulated platinum microelectrodes, which were inserted through the dura using a five-channel Eckhorn Microdrive (Thomas Recording; Eckhorn and Thomas, 1993). Electrodes were advanced while stimulating through the implanted pyramidal tract electrodes, until well isolated single units were encountered that responded antidromically to the pyramidal tract stimulation. The antidromic nature of the activation was confirmed by low jitter (<0.1 ms), sharp threshold, and a collision test (Baker et al., 1999).

Recordings from the reticular formation and spinal cord used U probe electrodes (Plexon), with 24 or 32 contacts equally spaced at 0.1 mm along the shaft (contact diameter, 15 µm; platinum/iridium alloy). For the reticular formation, U probes were driven into the brain to a depth of ∼15 mm using a standard stereotaxic manipulator, to just above the tentorium. Further fine positioning used a microdrive (Nan Instruments). The motor reticular formation was located relative to landmarks such as the abducens nucleus, as in our previous work (Soteropoulos et al., 2012; Zaaimi et al., 2018b). Spinal recordings targeted interneurons in the intermediate zone in segments C6–C8.

Once suitable single units had been located, recordings were made of activity during task performance. This used either a custom data acquisition system (based on hardware from National Instruments; for M1 in monkey V only) or a commercial system using digital headstages (Intan Technologies). Neural data, together with EMG recordings, task signals, and behavioral markers were digitized (sampling rate, 25 kHz) and saved to hard disk for offline analysis.

### Histology.

At the end of the spinal recordings, the animal was killed by an overdose of anesthetic (propofol, 25–30 mg/kg, i.v.) and then perfused through the heart with PBS followed by formalin fixative. Frozen parasagittal sections of the brainstem (thickness, 40 μm) were then cut, stained with cresyl violet, and photographed. A section ∼1.5 mm lateral to the midline for each animal was traced to delineate major landmarks, with reference to the similar tracings presented in the study by Sakai et al. (2009). The MRI scan taken early in the experiment was used to align this tracing to the stereotaxic coordinate system. Recording sites in the brainstem were then plotted onto the tracing, using the angles and penetration coordinates noted during the recording session. The location of the abducens nucleus was used as a reference to align histology and electrode locations: a small number of sites in the brainstem had elicited eye abduction movements after stimulation through the recording electrode.

### Data analysis.

High-pass-filtered (cutoff, 300 Hz) raw waveforms were first discriminated to the occurrence times of isolated single units. For M1 recordings using single-channel electrodes, this was achieved with manual cluster cutting using a custom program (Getspike, S.N.B.). Recordings from U probes were processed with the automated algorithm Mountainsort (Chung et al., 2017). Putative clusters were further processed with a semiautomated custom script to exclude artifacts and to combine separate clusters that resulted from a neural recording shifting from one contact to another because of recording instability. All accepted clusters had consistent action potential waveforms and interspike interval histograms with no counts in the first 1 ms corresponding to the absolute refractory period.

To ensure good task performance, trials were excluded if the elbow displacement trace exceeded 3° from the central hold position in the 2 s before the go cue (15% and 16% of trials, respectively, in monkeys V and U), and if the movement went in the wrong direction after the go cue (>3° toward flexion, when the target required extension, and vice versa; 32.6% and 16.3%, respectively, of trials in monkeys V and U). Overall, 60.7% and 74.5% of trials, respectively, were accepted using these combined criteria in monkeys V and U. Perievent time histograms (PETHs) were then compiled relative to the go cue (window, ±2 s; bin width, 10 ms) for each single unit and each target location; the modulation in firing rate was measured as the difference between the bin with the smallest and largest values. Interspike intervals for each trial were then randomly shuffled, using the process described in the study by Soteropoulos et al. (2012), and PETHs recompiled. This process was repeated 100 times. If the modulation of the PETH compiled from unshuffled data were larger than the 95th percentile of the shuffled, the modulation was considered significant. A cell was accepted as task modulated if any PETH compiled relative to the four different targets in response to go cue only showed significant modulation.

Cells typically modulated their firing with the location of the target; some fired most strongly for targets that required an elbow flexion, whereas others fired most for elbow extension. We measured the average firing rate over the 1 s period after the go cue for the two directions requiring most extreme flexion and extension; the target with the greatest rate was designated the preferred target. Before further processing of PETHs across cells, the targets were reordered to start with the preferred target, and then preserving the spatial arrangement so that the last target in the list was furthest from the preferred target (designated the nonpreferred target). Further analysis used the PETHs in this order, rather than relative to the actual spatial location of targets. A similar approach was used by Pruszynski et al. (2008) to combine reflexes across flexor and extensor EMG recordings.

For further analysis, PETHs were smoothed by a three-bin sliding window, in which each bin was replaced by the mean of its value and the one either side. PETHs were then averaged across cells. Differences between PETHs after go cue only, and for go cue with loud sound, were assessed bin-by-bin across cells using a paired *t* test. The *p* values were adjusted using the Benjamini–Hochberg correction for multiple comparisons to take account of testing many bins (Benjamini and Hochberg, 1995).

We wished to calculate a measure of how much the population activity differed between the preferred and nonpreferred target. Assume the raw, unsmoothed PETH in cell *i* to the preferred target contains the number of spike counts *X_i_*(*j*) in bin *j*, accumulated over *N_i_* trials of the task. Because spike counts approximately follow a Poisson counting process, the variance of *X_i_*(*j*) will be *X_i_*(*j*). We wish to convert these raw PETHs into firing rates (in hertz), removing the dependence on the arbitrary number of trials recorded and on the bin width. The estimate of mean rate µ*_i_*(*j*) (in hertz) and its variance σ*_i_*^2^(*j*) will be as follows:

$$\mu_{i}(j)=\frac{X_{i}(j)}{N_{i}\Delta },$$

$$\sigma_{i}^{2}(j)=\frac{X_{i}(j)}{N_{i}{}^{2}\Delta^{2}},$$

where Δ is the bin width (here 10 ms). Assuming *Y_i_*(*j*) is the corresponding PETH to the nonpreferred target, accumulated over *M_i_* trials, the difference between the firing rates of the entire population of *n* recorded cells between the two targets is as follows:

$$D\left(j\right)=\overset{n}{\underset{i=1}{\sum }}\frac{X_{i}(j)}{N_{i}\Delta }-\frac{Y_{i}(j)}{M_{i}\Delta }.$$

The variance of *D*(*j*) is as follows:

$$S\left(j\right)=\overset{n}{\underset{i=1}{\sum }}\frac{X_{i}(j)}{N_{i}{}^{2}\Delta^{2}}+\frac{Y_{i}(j)}{M_{i}{}^{2}\Delta^{2}}.$$

As for the PETHs described above, we generated smoothed estimates *D′*(*j*) and *S′*(*j*) by summing over three adjacent bins, as follows:

$$D^{\prime }\left(j\right)=D\left(j-1\right)+D\left(j\right)+D\left(j+1\right),$$

$$S^{\prime }\left(j\right)=S\left(j-1\right)+S\left(j\right)+S(j+1).$$

Finally, we formed a *z* score as follows:

$$z\left(j\right)=\frac{D\prime (j)}{\sqrt{S\prime (j)}}.$$

This will be approximately normally distributed, with mean of 0 and an SD of 1 on the null hypothesis that the population firing rate is the same for the preferred and nonpreferred target. Values of |*z*| > 1.96 indicated a significant difference between the targets (*p* < 0.05), with greater |z| values indicating greater differences.

It was of additional interest to determine whether the difference in firing between the preferred and nonpreferred targets was altered by the loud sound. We therefore calculated the following:

$$\zeta \left(j\right)=\frac{z^{\text{sound}}\left(j\right)-z^{\text{no sound}}(j)}{\sqrt{2}},$$

where the normalization factor of $\sqrt{2}$ ensured that ζ(*j*) will be normally distributed, with a mean of 0 and an SD of 1, on the null hypothesis that the difference in firing between targets was unchanged by the sound.

Analysis of EMG was not possible for all recording sessions, because of technical failures of implanted wires throughout the recording period. We therefore confined analysis to early sessions, when EMG signals remained of high quality, and measured the response onset latency after the go cue. The SD of the unrectified EMG on each trial was measured from 200 ms before to 75 ms after the go cue. The median and SD of these values were found across trials; trials exceeding the median + 2 SDs were excluded from this part of the analysis, as they were likely to contain artifacts. Starting 75 ms after the go cue, the first EMG sample to exceed 5× the baseline SD was taken as the response onset. For display purposes, an average of rectified EMG compiled across trials was computed separately for trials with and without a loud sound cue. Mean activity in nonoverlapping 10 ms bins was compared between these averages using *t* tests; determination of which bins were significantly different used the Benjamini and Hochberg (1995) correction for multiple comparisons.

### Computational model

The experimental data revealed how neural activity in different motor centers modulated during task performance, and how this modulation was altered by the loud sound. We used a computational model to understand the consequences for motor output of these patterns of neural modulation.

**Figure 2.**
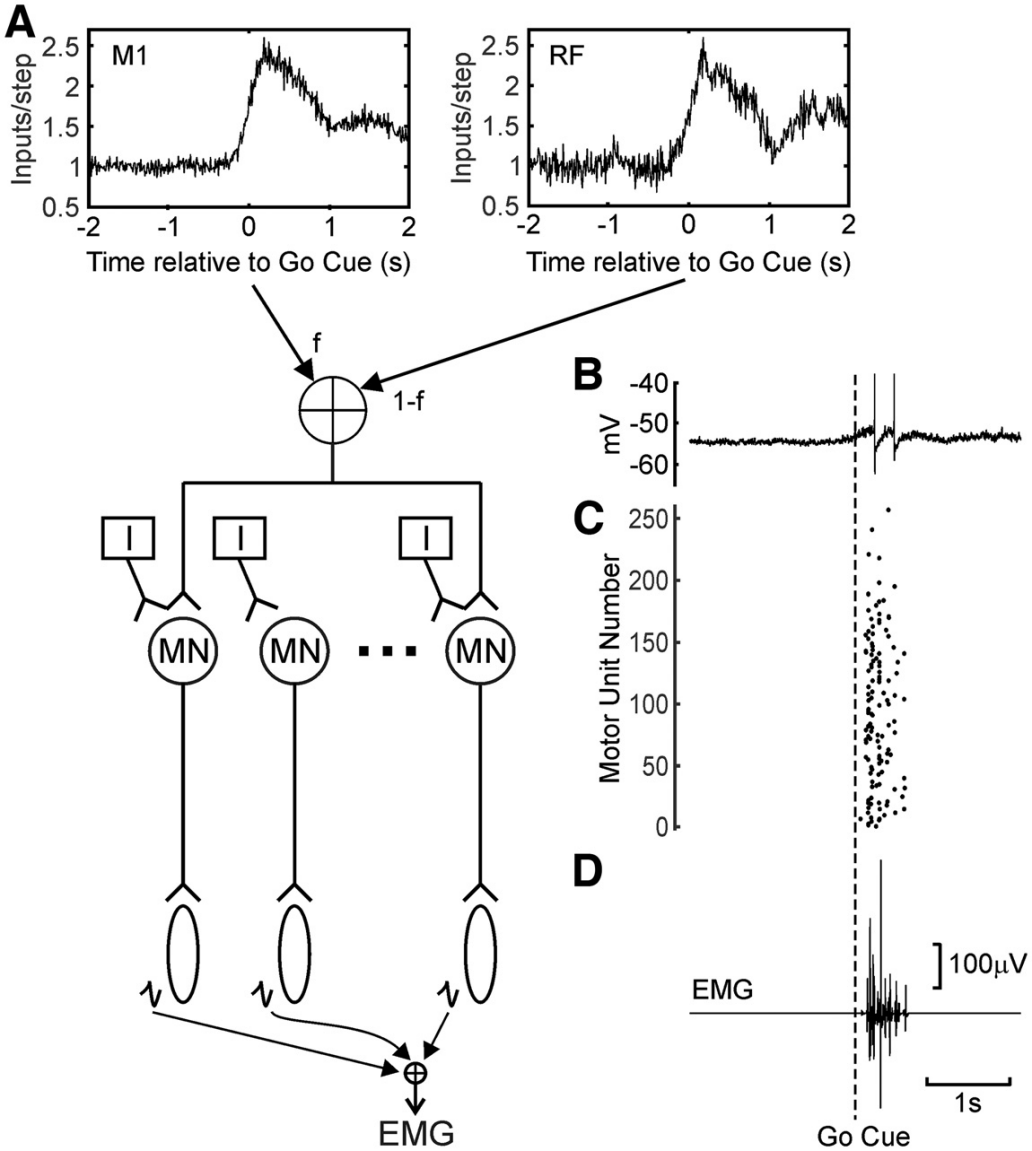
Computational model. ***A***, Experimentally determined average rate profiles for populations of cells from M1 and RF were scaled to have the same background and peak rates, and then mixed with a mixing factor *f*, which determined the ratio of M1 to RF drive. A pool of motoneurons received inputs modulated with this rate profile. ***B***, Whenever a motoneuron fired a spike (example membrane potential), it generated a motor unit action potential. ***C***, ***D***, The population activity over the motoneuron pool (***C***, rasters) summed to give a simulated EMG (***D***).

The model was based on previous publications from this group (Baker and Lemon, 1998; Williams and Baker, 2009a, b), and is illustrated in Figure 2*A*. A pool of 377 motoneurons received rate-modulated input. Each motoneuron received input from the following two sources: one specific to that motoneuron; the other common across the pool. In each 0.2 ms time step of the stimulation, the number of excitatory inputs to a given motoneuron *e_i_* was determined by the following:

$$e_{i}=\text{Poiss}\left(0.8\lambda \right)+C,$$

C=Poiss(0.2λ).

Where λ was the current rate of input per time step, and Poiss(*x*) indicates drawing a random number from a Poisson distribution with mean equal to *x*. *C* was chosen once per time step, to reflect the common input to all motoneurons. This procedure led to inputs which modulated according to λ for all motoneurons, but which had 20% common fluctuation across the pool. The firing rate λ was determined by summing together the rate modulation of pyramidal tract neurons (PTNs) from M1 (λ*_M1_*) and cells from the reticular formation (λ_RF_), according to the following:

$$\lambda \left(t\right)=f\lambda_{\text{M1}}\left(t\right)+(1-f)\lambda_{\text{RF}}\left(t\right),$$

where the parameter *f* determined the relative importance of M1 and reticular formation drive to the motoneurons. The profiles of λ_M1_ and λ_RF_ were determined from the experimentally measured PETHs, normalized to have a baseline rate of 5 kHz, and a peak rate of 13 kHz. These values were determined from preliminary simulations to generate realistic EMG responses from a resting baseline.

Motoneurons were simulated using the conductance-based model developed by Booth et al. (1997) and were used in previous work from this laboratory. The variation of motoneuron parameters across the pool, which leads to orderly recruitment according to the size principle (Henneman et al., 1965; Zajac and Faden, 1985), was modeled heuristically by changing the fraction of input from the dendritic tree, as described in the study by Williams and Baker (2009a). Whenever a motoneuron generated an action potential (Fig. 2*B*,*C*), this led after a delay in the replay of its characteristic motor unit action potential. These summed linearly to generate the EMG recording (Fig. 2*D*).

## Results

### The StartReact effect: reaction time shortening by a loud sound in monkey

**Figure 3.**
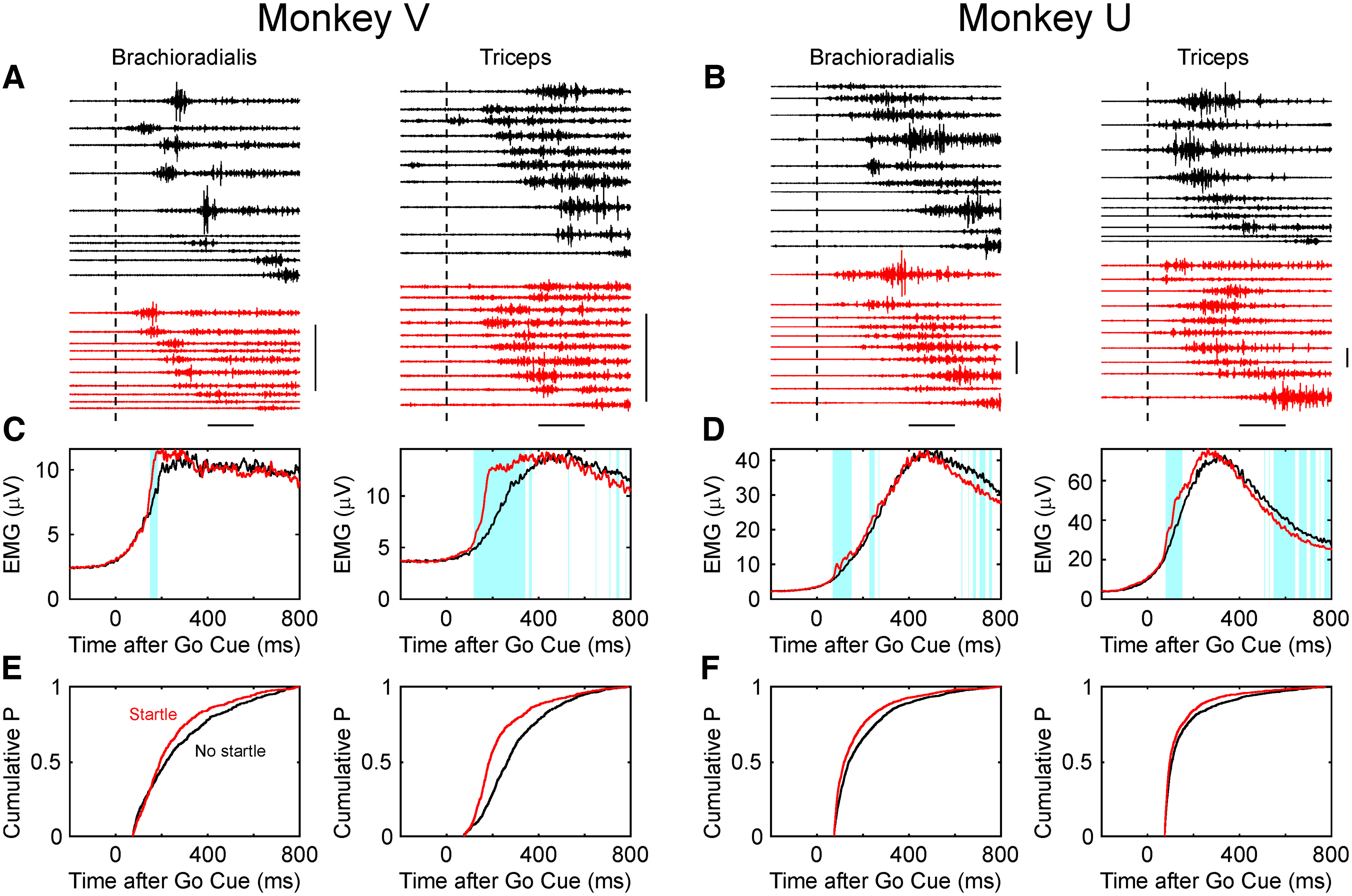
The StartReact effect in monkey EMG. ***A***, Single sweeps of EMG from the brachioradialis (left) and triceps (right) muscles, from monkey V. Traces have been aligned on the go cue, for the instructed target nearest to or farthest from the monkey, for brachioradialis and triceps muscles, respectively. Black traces, The response following a go cue only; red traces, following a go cue combined with a loud sound. Dotted line shows the time of the cue. The 10 traces have been selected to represent the 5th, 15th, and 25th up to 95th percentile of the reaction time distribution. Calibration: 1 mV, 200 ms. ***B***, As ***A***, but for monkey U. ***C***, Average of rectified EMG from monkey V. Blue shading shows sections 50–800 ms after the go cue where EMG activity differed significantly between responses to go cue only and go cue with loud sound. ***D***, As in ***C***, but for monkey U. ***E***, Cumulative distribution of reaction times measured from each single trial of the data that generated the averages in ***C***. ***F***, As for ***E***, but for monkey U corresponding to averages in ***D***. Measures compiled from between 628 and 1422 trials of the task.

Figure 3 presents data showing that the StartReact effect can be observed in monkeys. Figure 3, *A* and *B*, presents illustrative single sweeps of EMG, and Figure 3, *C* and *D*, presents averages of rectified EMG, triggered by the onset of the go cue. For the brachioradialis muscle, trials were used where movement to the target closest to the monkey was instructed; this required flexion around the elbow joint. For the triceps, trials to the target farthest away from the monkey were used, which required elbow extension. EMGs showed a clear increase after the go cue, corresponding to the initiation of movement (black traces); when a loud sound was given at the same time as the go cue, there was a subtle increase in early activity (red traces). This increase was significant for both muscles in each monkey (Fig. 3*C*,*D*, blue shading).

Figure 3, *E* and *F*, quantifies this effect at the level of single trials. The onset latency of EMG activity above baseline was measured and was plotted as a cumulative probability distribution across all available trials. The cumulative curves for trials with loud sound were shifted to earlier onsets; this is the StartReact effect. Reaction times were measured as follows (given as time without and with loud sound, followed by difference Δ and a *p* value for statistical comparison with *t* test): monkey V: brachioradialis muscle, 311/280 ms, Δ = 31 ms, *p* = 0.0017; triceps, 323/278 ms, Δ = 45 ms, *p* = 1.6 × 10^−10^; monkey U: brachioradialis muscle, 223/183 ms, Δ = 40 ms, *p*= 3.6 × 10^−13^; triceps, 184/154 ms, Δ = 30 ms, *p* = 5.2 × 10^−9^.

### Available single-unit recordings

Table 1 gives the number of cells recorded from each area in each monkey. In M1, we searched for cells that were identified as corticospinal neurons by antidromic activation after stimulation of the pyramidal tract (PTNs). Some unidentified cells were encountered; the numbers are reported in Table 1, but further analysis used only the identified PTNs. The antidromic latency of the PTNs was 2.1 ± 1.2 ms (mean ± SD; range, 0.7–6.5 ms). This is comparable with previous reports and suggests that the recordings were heavily biased to the corticospinal cells with the fastest conducting axons (Kraskov et al., 2019). PTNs were further classified based on the electrode depth after the first cells were detected, providing an estimate of whether they were in the bank of the central sulcus or on the surface (corresponding to new M1 and old M1, respectively, in the terminology of Rathelot and Strick, 2009). Most cells so classified as located in new M1 were recorded in monkey U.

**Table 1.** Recording database

Area	Monkey V	Monkey U	Total
M1, unidentified cells	23 (7)	73 (32)	96 (39)
M1, PTNs	45 (24)	114 (71)	159 (95)
Superficial (Old M1)	43 (22)	81 (48)	124 (70)
Deep (New M1)	2 (2)	33 (23)	35 (25)
RF	84 (34)	286 (107)	370 (141)
Gi	84 (34)	134 (50)	218 (84)
PnC/PnO	0 (0)	152 (57)	152 (57)
SC	73 (0)	53 (34)	126 (34)

The number of cells recorded in each category is followed in brackets by the number that modulated significantly with the task.

**Figure 4.**
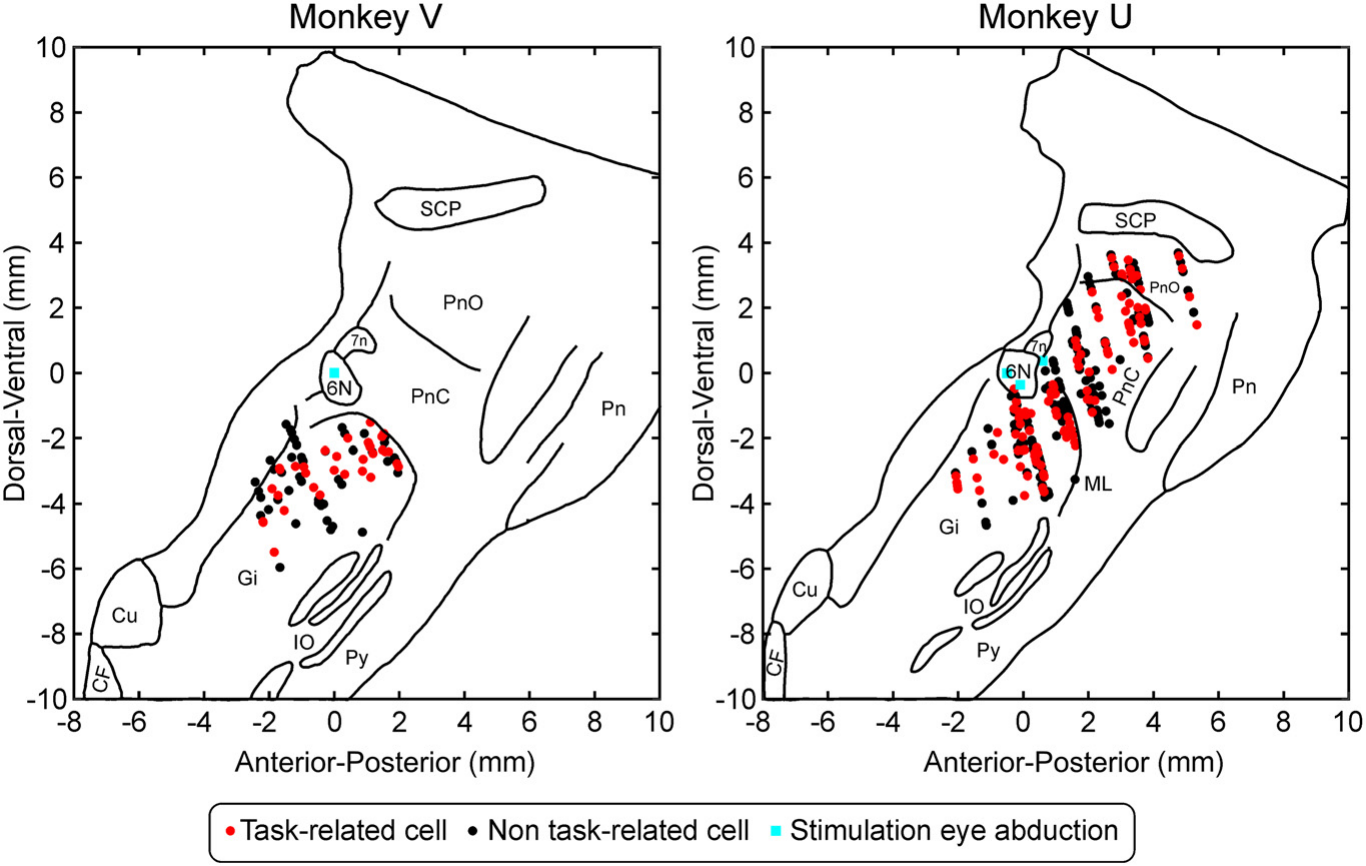
Estimated location of brainstem recording sites. For each monkey, a tracing is shown of a parasagittal section of the brainstem, at ∼1.5 mm lateral to the midline. The orientation of the brainstem has been rotated to align with standard stereotaxic coordinates, based on MRI scans taken in a sitting posture. Anterior–posterior and dorsal–ventral coordinates are expressed relative to the interaural line, with positive numbers indicating anterior and dorsal, respectively. Black and red dots mark the estimated location of recorded single neuron activity; the color shows if the cell was significantly modulated by the task (red) or not (black). Cell locations have been aligned to sites where stimulation generated clear ipsilateral eye abduction, corresponding to the abducens nucleus (blue dots). 6N, Abducens nucleus; 7n, facial nerve; CF, cuneate fasciculus; Cu, cuneate nucleus; IO, inferior olive; Pn, pons; Py, pyramidal tract; SCP, superior cerebellar peduncle.

In the reticular formation, the location of recorded cells within the brainstem was reconstructed using penetration coordinates. Recording sites were aligned to tracings of postmortem parasagittal sections in each monkey, using sites where stimulation produced eye abduction to align to the abducens nucleus, which is a key landmark (Fig. 4). Many more cells were recorded from monkey U than from monkey V; these covered a wider rostrocaudal extent. All cells within monkey V seemed to come from the nucleus gigantocellularis (Gi), whereas in monkey U recordings were approximately evenly split between Gi and the more rostral pontine reticular nuclei (PnC/PnO). Cells with significant task modulation (red dots) were interspersed with non-task-related cells (black dots) in both monkeys.

In the spinal cord, although more cells were recorded in monkey V, none of these were significantly modulated with the task; the usable data for the spinal cord thus came entirely from monkey U.

### Task-related modulation of neural activity is altered by loud sound

To investigate how cells modulated their activity with task performance, we compiled perievent histograms relative to the go cue. Some cells fired most with the target closest to the monkey (requiring elbow flexion), whereas others fired most for the target furthest away (elbow extension). This is in agreement with previous reports of movement-related activity tuning in M1 (Georgopoulos et al., 1986), RF (Siegel and Tomaszewski, 1983; Buford and Davidson, 2004), and SC (Fetz et al., 1996). This task modulation created problems if averaging across cells was performed according to target, as changes between targets would be smoothed out. To compensate for this, we measured the average firing rate over the 1 s period after the go cue, for the two most extreme targets, and designated the target with the highest rate as the preferred target. Rather than order targets by their physical location (e.g., nearest to furthest away), we then reordered the targets for each cell so that the preferred target was always the first in the list. Note that this procedure did not change the sequential arrangement of targets—simply the order, either from most extreme flexion to most extreme extension or vice versa.

**Figure 5.**
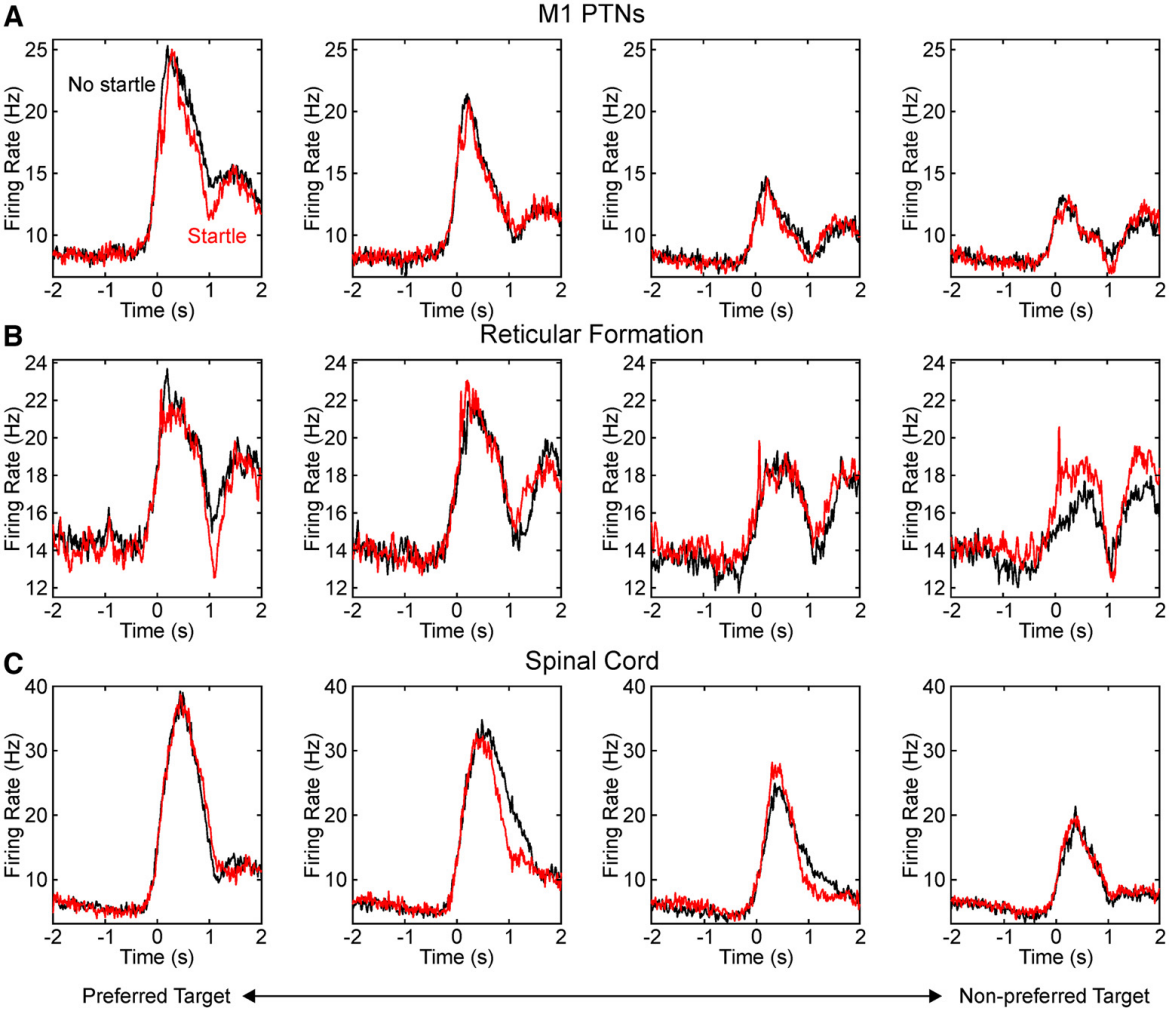
Average perievent histograms. Traces show the average cell firing rate, measured across all cells in the stated population, which were significantly modulated with the task. Firing has been aligned to the go cue at time 0. For each cell, we determined whether the target nearest to or furthest away from the monkey gave the greatest firing rate. The left column shows averages for the preferred target, and subsequent columns show averages for the immediately adjacent target. Black traces, The response following a go cue only; red traces, go cue combined with loud sound. ***A***, For antidromically identified PTNs from M1. ***B***, For cells in the RF. ***C***, For interneurons in the cervical enlargement of the spinal cord.

Figure 5 presents perievent histograms for the preferred target (left plots), nonpreferred target (right plots), and those in between (middle plots). The histograms have been averaged across the different populations available. In each case, black curves reflect activity to the go cue alone; red when the go cue was combined with a loud sound. In all areas, the activity began to rise above baseline before the go cue. This presumably reflects preparation for the forthcoming instructed movement. The broad profile of rate modulation was remarkably similar between the three different brain and spinal cord areas. This was only subtly altered by the loud sound. We would expect that this broad similarity of rate modulation should lead to similar movements being produced following a loud sound and after less arousing stimuli, as has been previously reported (Valls-Solé et al., 1999; Dean and Baker, 2017) and as also seen in the similar EMG activation patterns observed in this study (Fig. 3).

**Figure 6.**
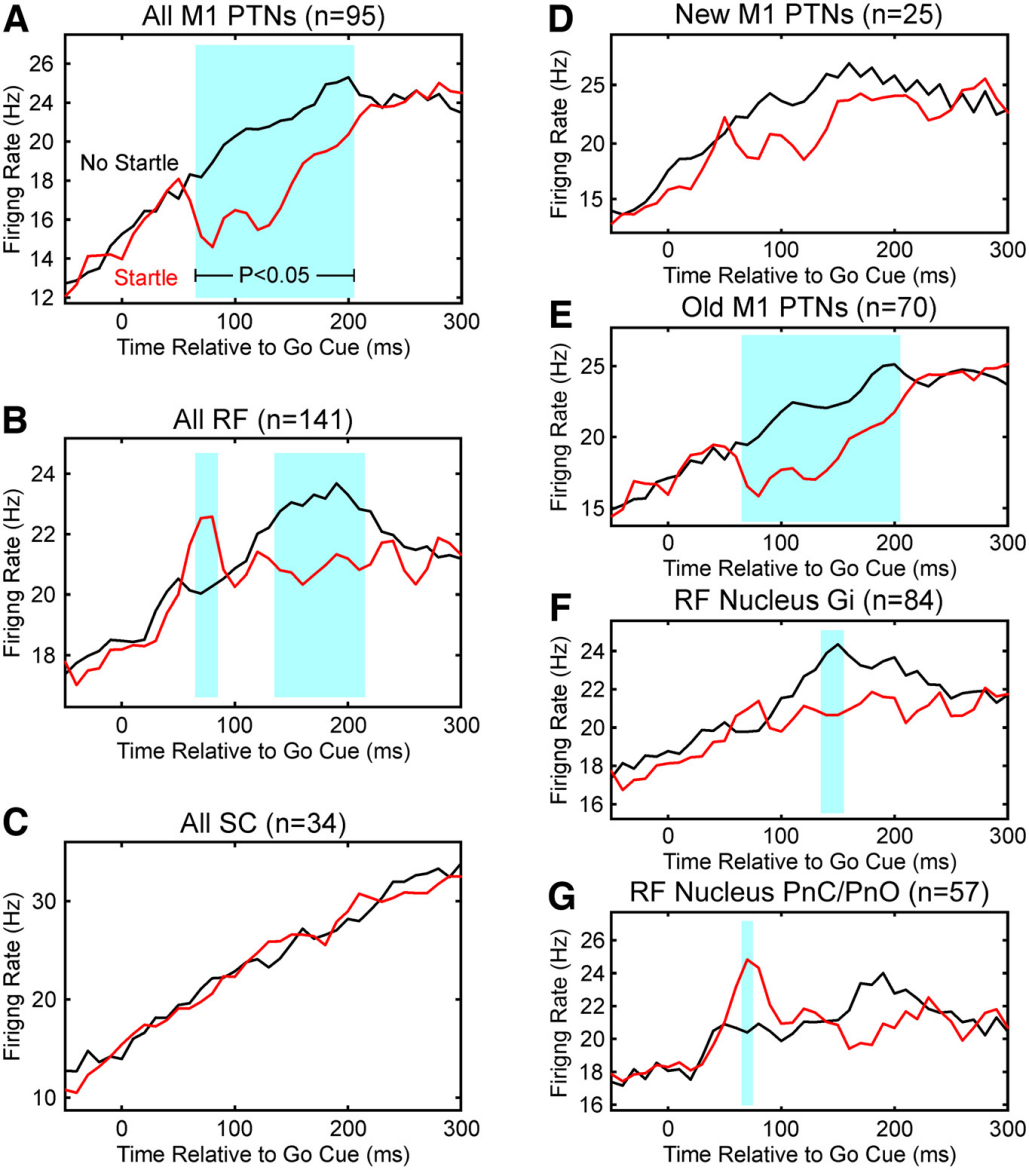
Average perievent histograms on an expanded timescale. Traces show the perievent histograms from Figure 5 for preferred target, on an expanded timescale to illustrate the differences produced by the loud sound more clearly. ***A***, For M1 PTNs. ***B***, For reticular formation. ***C***, For spinal cord. ***D***, For M1 PTNs in the bank of the central sulcus (New M1), ***E***, For M1 PTNs on the gyrus (Old M1). ***F***, For RF cells estimated to lie within the Gi. ***G***, For RF cells estimated to lie in the pontine reticular nuclei (PnC/PnO). Blue shading indicates bins where activity after loud sound (red) was significantly different from after the visual go cue only (black).

However, the loud sound did produce a small but important difference in the neural activity at short latencies, which can be better appreciated on the expanded timescale of Figure 6 (for preferred targets only). For cells in M1, firing was transiently significantly suppressed by the loud sound (Fig. 6*A*), for bins 70–200 ms after the cue (blue shading). By contrast, for the RF cells (Fig. 6*B*), there was a brief increase in rate from 70 to 80 ms, followed by a significant decrease (140–210 ms). For cells from the spinal cord, there were no significant changes generated by the loud sound (Fig. 6*C*).

Figure 6, *D* and *E*, shows results for M1 PTNs, separated by whether the recording was within the bank of the central sulcus (New M1) or the gyrus (Old M1). A clear suppression was produced by the loud sound for both populations, but only reached significance for Old M1 (Fig. 6*E*, blue shading) likely because of the small number of cells available from New M1. We measured the mean difference in firing rate between trials with and without the loud sound, over the region marked in blue in Figure 6*A* for each cell. The suppression was 3.9 ± 8.7 Hz for Old M1, and 3.2 ± 8.5 Hz for New M1 (mean ± SD), which was not significantly different (*p* = 0.72, *t* test).

For the reticular formation, we separated the cells according to their estimated location within the brainstem (Fig. 4). The early facilitation of activity only reached significance for the PnC/PnO population (Fig. 6*G*). When measured over the first blue-shaded region marked in Figure 6*B*, the facilitation was 1.4 ± 9.5 Hz for Gi, and 3.9 ± 11.4 Hz for PnC/PnO, which was not significantly different (*p* = 0.15, *t* test). The late suppression only reached significance for the Gi population (Fig. 6*F*). Measurements over the second blue-shaded region in Figure 6*B* yielded a suppression of 2.4 ± 8.0 Hz for Gi, and 2.1 ± 8.9 Hz for PnC/PnO (also not significantly different, *p* = 0.80, *t* test).

### Neural representation of different target movements

**Figure 7.**
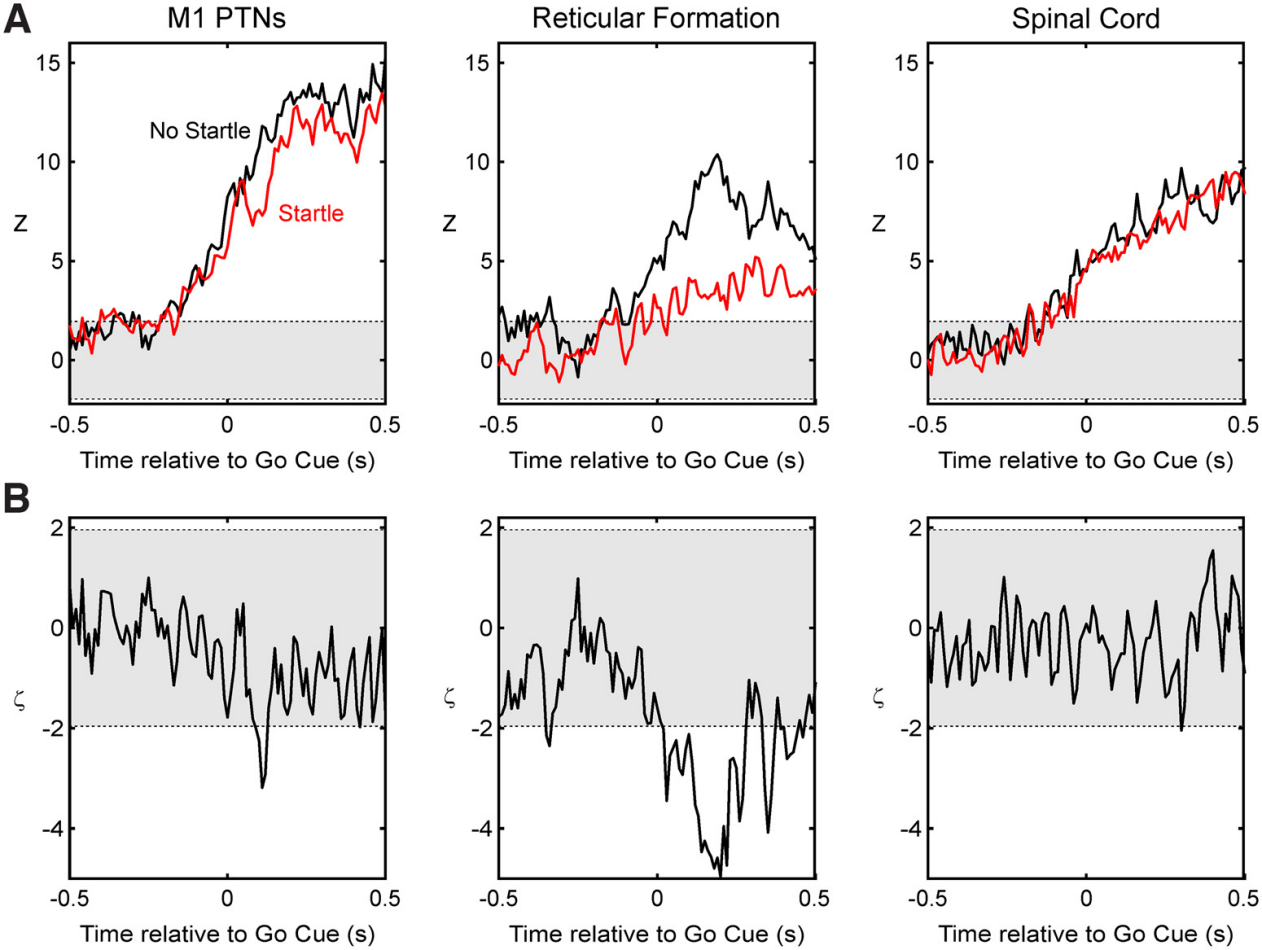
The *z* score analysis. ***A***, Population *z* score for each area, showing how activity differed between preferred and nonpreferred targets. Gray shading indicates −1.96 < *z* < 1.96, corresponding to the 95% confidence limit on activity that is no different between targets. Different lines show results for trials with go cue only (black), and after a loud sound (red). ***B***, Difference between the corresponding red and black traces from ***A***, scaled to remain as a *z* score (measure defined as ζ in Materials and Methods). Positive values above the confidence limits (also gray shading) indicate that the loud sound significantly increased the difference in cell activity between the two targets; negative values indicate that the loud sound decreased this difference.

Neural firing is different for movements toward or away from the preferred direction of a cell. This difference forms the basis for neural encoding of movement. In the previous section, we showed that a loud sound can modify firing rates, but it is not clear whether this represents merely the linear sum of facilitation or suppression on top of unchanged task-related activity, or whether the neural representation of the movement is altered, so that firing rates become more or less similar for different movements after the loud sound. To address this, we calculated a *z* score for each cell (see Materials and Methods). This measured how different firing was between the preferred target and the nonpreferred target; the *z* score will be close to zero if firing is similar between the two targets. Results are shown for M1, RF, and SC cell populations in Figure 7*A*. For all three populations, the *z* scores rose above the significance limits (gray shading) before the go cue, reflecting preparatory activity in response to the instructional cue. For M1, there was a reduction in discriminability between the two targets caused by the loud sound, at the same time as the suppression of cortical activity. The difference between these two *z* scores (denoted ζ in Materials and Methods) is shown in Figure 7*B*; this confirmed that the reduction exceeded that expected by chance (also gray shading). A more sustained reduction in ζ was also seen in RF, although the loud sound initially had the opposite effect of increasing neural activity. Finally, the coding of target within spinal cord interneurons was unaffected by the loud sound, as expected given the lack of changes in rate seen in Figure 6*C*. It should be emphasized, however, that, although changes for M1 and RF following the loud sound could be detected statistically, the coding of target location remained significant for both populations (Fig. 7*A*). The change was especially small for M1: at the point of maximum difference between the curves in Figure 7*A* (110 ms after go cue), the *z* score after loud sound was 7.3. This still corresponds to a very high level of discrimination between the two targets.

### A computational model reveals the impact of loud sound-induced firing changes for behavior

In the experimental studies reported here, we have directly measured changes in neural firing rate in key motor centers. However, it is unclear what the impact of these changes will be for behavior, and in particular for reaction time, which is the usual outcome measure in human studies of the StartReact effect. To examine this, we used a computational model, as described in Materials and Methods (Fig. 2). This allowed rate modulation profiles of inputs to a motoneuron pool to be converted to overt motor output, via a realistic conductance-based model, which incorporated the nonlinear nature of the motoneuron response.

**Figure 8.**
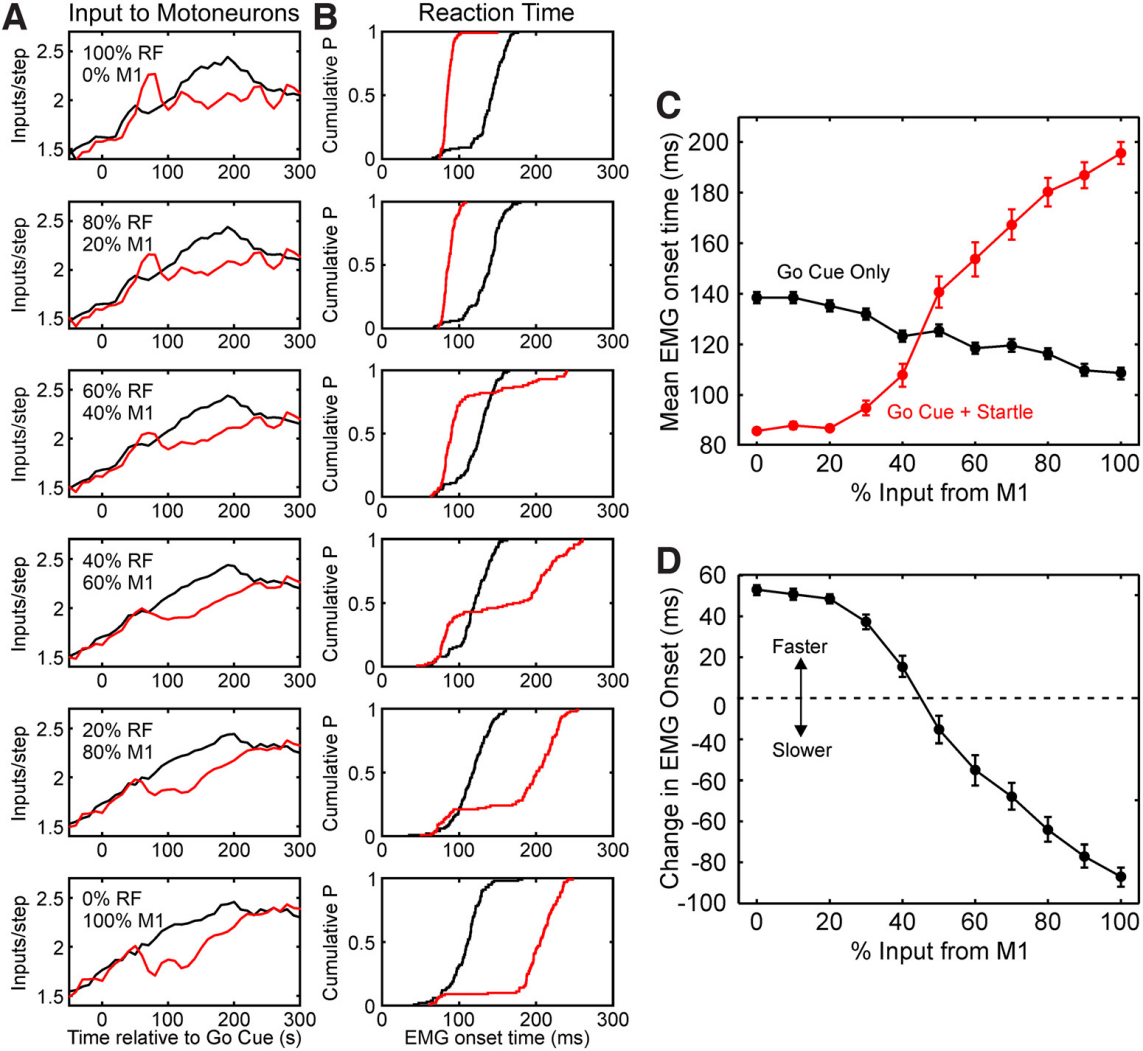
Computational model results. ***A***, Time profile of synaptic inputs to the motoneuron pool per 0.2-ms-long time step. These plots have been generated by scaling the experimentally determined profiles in Figures 5 and 6 in response to the preferred target, and then mixing activity from RF and M1 in the relative proportions shown. Black, Input for trials following the visual go cue only; red, when the cue is combined with a loud sound. ***B***, Cumulative probability distribution plots of reaction time, measured from simulated EMG, for the different profiles of input to the motoneuron pool shown in ***A***. ***C***, Variation in reaction time with the proportion of input derived from M1, for trials following a go cue only (black) or after a go cue and loud sound (red). ***D***, Differences between reaction times shown in ***C***, calculated so that positive differences indicate a faster reaction time, and negative differences indicate a slower reaction time, after a loud sound. Note that only if <50% of input to the motoneuron pool comes from M1 is the reaction time shortened by a loud sound, as seen experimentally (Fig. 3). Points in ***C*** and ***D*** show the mean and SEM, calculated over 100 simulated trials for each condition.

Figure 8, *A* and *B*, presents results from six simulations, in which the input to the model was generated from varying the mixture of M1 and RF activity, as described in Materials and Methods. The top row in Figure 8, *A* and *B*, shows the situation when only the RF drove movement; the bottom row, when only M1 drove movement; the middle rows, intermediate cases where motoneuron drive was contributed by both M1 and RF to varying degrees. Before generating these rate profiles, the experimentally measured rates were scaled to have the same baseline and peaks. This avoided small differences in absolute firing rates in our experimental datasets producing trivial differences in motor output; instead, we focus on the effect of the shape of modulation and the changes produced by a loud sound.

Figure 8*A* shows the rate modulation profiles. These were very similar for go cue only (black). It mattered little whether drive came from the population of M1 or RF cells, the temporal profile of rate change across a trial of the task was very similar. However, the profile after a loud sound (red) was altered depending on the source of drive to motoneurons. If drive came only from RF, the loud sound produced a sharp increase in rate, whereas if drive came only from M1, there was a fall in rate (Figs. 5, 6, as expected).

Figure 8*B* plots the cumulative distribution of reaction time, measured from the simulated EMG traces in exactly the same way as from the experimental EMG traces of Figure 3. It should be noted that the modeled reaction time distributions were less variable than those seen experimentally: the model always produced reaction times <300 ms. By contrast, 36% and 39% of reaction times after go cue alone were >300 ms in monkey V for the brachioradialis and triceps muscles, respectively; the corresponding figures for monkey U were 19% and 12% (Fig. 3*E*,*F*). It is perhaps unsurprising that our relatively simple modeling approach could not replicate precisely all aspects of the experimental data. In particular, the input to the model modulated according to the trial-averaged population firing rates described above. Instantaneous spike counts were determined from Poisson counting statistics with this fixed rate profile. In reality, rate modulation varies from trial to trial, and this fluctuation is correlated between different cells (Baker et al., 2001). It was impossible to model this in a principled way, as the cell population was not recorded simultaneously in this study, but it seems probable that this may partly underlie the reduced reaction time variability in the modeled responses. Nevertheless, the model did allow us to examine how different mixtures of descending input to motoneurons would modulate reaction time. When motoneuron drive was dominated by the RF, a broad distribution of reaction times in response to the go cue only was replaced by a narrow distribution of early reaction times following a loud sound. By contrast, when drive was dominated by M1, reaction times became more variable and on average slower following the loud sound.

Figure 8*C* quantifies these changes in reaction time as a function of the mixture of M1 and RF activity, shown on the abscissa as a percentage. Although rate profiles for the go cue-only trials were broadly similar for different sources of drive to movement, there was a small but consistent change in reaction time as the mixture changed (Fig. 8*C*, black trace), with reaction time 30 ms shorter for input 100% from M1 than for input 100% from RF. For the trials in response to a loud sound, there was a much larger effect of changing the mixture in the opposite direction: reaction time was 110 ms faster with input solely from RF compared with input solely from M1.

The difference between the two reaction time curves is plotted in Figure 8*D*. Reaction time was faster following loud sound if ≤40% of input to motoneurons came from M1. Results comparable to the experimentally measured StartReact effect could thus only be reproduced if the majority of input came from RF. By contrast, for higher proportions of input from M1, a loud sound slowed the reaction time—the opposite of experimental findings. It is notable that there was a floor effect, so that reaction time was not further decreased if the proportion of M1 input fell to <20%. By contrast, reaction time continued to rise as the proportion of M1 input increased, all the way to 100%.

## Discussion

### Mechanisms of the StartReact effect

The present study addresses the generation of the StartReact effect using direct recordings from monkeys.

One previous hypothesis suggested that StartReact arises from cortical facilitation by the reticular activating system, which itself is stimulated by a loud sound (Carlsen et al., 2012). Contrary to this idea, we found that cortical activity was briefly suppressed by sound; this is consistent with the suppression of muscle responses to cortical stimulation by loud sounds reported in humans (Furubayashi et al., 2000). In simulations where motoneuron drive came only from the CST, reaction times were delayed, not accelerated. StartReact cannot therefore be explained by cortical circuits alone.

An alternative hypothesis is that motor programs—the specification of which muscles must be activated, and at what times, to achieve the movement goal—are downloaded from cortex to brainstem, and then rapidly triggered by the loud sound (Valls-Solé et al., 1999; Rothwell, 2006). This idea could account for the close similarity of voluntary and startle-evoked movements (Valls-Solé et al., 1999; Dean and Baker, 2017; Ossanna et al., 2019). However, in our recordings loud sound did not ignite a rapid movement by bringing forward the task-dependent modulation of firing in the brainstem. Instead, activity became slightly less selective after the loud sound (Fig. 7). Far from the motor program being triggered early, it was partially corrupted at both cortical and brainstem levels by superimposition of the response to sound.

The present findings offer a different interpretation of StartReact. Critically, our results suggest that normally sluggish reaction time arises because motoneurons traverse a slowly rising trajectory toward spike threshold. Firing rates in all areas began to rise even before the go cue, presumably reflecting anticipatory preparation for the forthcoming movement. However, temporal summation meant that motoneurons only spiked after a delay. This is consistent with early reports that PTNs are activated ∼100 ms before movement onset (Evarts, 1966). The startle stimulus boosted reticular formation activity, and thereby accelerated the rise to threshold of motoneurons. This boost was relatively nonselective—it actually led to reticular activity discriminating less well between movements to different directions. However, there was still sufficient selectivity to ensure that the correct motoneuron pools were activated (Fig. 7*A*, high *z* scores). Our findings emphasize that the motor program continues to play out almost as usual during startle trials. There is not a shift from cortex to brainstem in the origin of the command, which specifies which movement is to be produced. Rather, a relatively nonselective additional drive is superimposed on the usual command (Maslovat et al., 2014, 2015). This shortens reaction time; because the command remains largely intact, movements retain their key characteristics, as reported previously (Valls-Solé et al., 1999; Dean and Baker, 2017).

The facilitation of activity in the reticular formation was brief (70–80 ms after the cue). However, because motoneurons integrate their inputs to reach firing threshold, this would be capable of affecting reaction times even after the augmented input had passed. The facilitation was followed by a later suppression of the reticular formation (140–210 ms after the cue), at which time the CST cells were also suppressed. It might be expected that this ubiquitous late suppression would lead to an extra response delay following loud sounds on trials with slow reaction times, but this did not seem to occur (Fig. 3*E*,*F*). Our data cannot directly address this discrepancy, but it should be remembered that some spinal cord interneurons receive input from both RST and CST (Riddle and Baker, 2010). The resulting oligosynaptic effects on motoneurons can show nonlinear interactions (Dyson et al., 2014), which were not incorporated into our model.

### Relative drive to motoneurons from corticospinal and reticulospinal tracts

Startle had opposite effects on early population firing in the cortex and reticular formation. Our modeling predicted that this would lead to opposite effects on reaction time. This fortuitous set of circumstances allowed us to conclude that the majority of the motoneuron drive is likely to arise from the RST, not the CST. We did not observe substantial shifts in relative firing between RST and CST following the loud sound; the changes were transient and quite subtle. The only interpretation consistent with the experimental data is that the RST provided the most drive, not only after the loud sound but also for movements following a nonstartling cue.

This may appear to be a surprising result, but it is consistent with other findings. Net drive to motoneurons is related to the following three factors: the firing rate, the number of fibers, and the strength of synapses. The profiles of rate modulation and peak firing rates were similar between PTNs and RF cells for the preferred target (Fig. 5*A*,*B*), as we have also previously reported for a finger movement task (Soteropoulos et al., 2012). It should be noted, however, that while we were able to identify PTNs antidromically, this was not possible for RST cells. It is conceivable that identified RST cells might have shown a greater modulation than the unidentified population in our sample.

The number of fibers providing input to motoneurons, and the strength of individual synaptic connections, are difficult to assess directly. In anesthetized animals, we previously estimated that compound EPSPs from the monosynaptic corticomotoneuronal connections were approximately five times larger than monosynaptic and disynaptic inputs from the RST (Riddle et al., 2009; Zaaimi et al., 2012). However, measurements under anesthesia, from gross stimulation of a whole motor tract, can significantly underestimate the contribution of oligosynaptic linkages. This is because interposed interneurons may be rendered less excitable by anesthesia. Excitation can also be subject to cancellation by unphysiological coactivation of feedforward inhibition (Alstermark et al., 1999). During the performance of a motor task, convergence of CST and RST inputs onto spinal cord interneurons (Riddle and Baker, 2010) will by contrast facilitate these indirect pathways.

Other quantitative results also support the idea that monosynaptic corticospinal inputs form the minority of motoneuron drives. In our computational model, the peak input firing rate required to generate a phasic EMG burst was 13 kHz. This compares with the peak seen in PTNs of ∼25 Hz (Fig. 5*A*), suggesting that ∼520 such inputs would be required. Following electrical stimulation of the macaque pyramidal tract, compound EPSPs in upper limb motoneurons are 0.7–3.5 mV (Fritz et al., 1985; Riddle et al., 2009). Unitary corticomotoneuronal EPSP amplitude is 25–120 μV (Asanuma et al., 1979). Dividing these amplitudes yields estimates of 6–140 corticomotoneuronal fibers projecting to a single motoneuron. Even at the upper end of this range, this falls far short of the estimate of 520 inputs required. This simple calculation may be subject to errors: it assumes that pyramidal tract stimulation activates all PTNs synchronously; in fact, some fibers may not be activated because of adverse placement relative to the stimulating electrode. It also assumes perfect summation of unitary EPSPs to form the compound EPSP. This will only occur for the fastest CST axons; for the many slow fibers (Firmin et al., 2014), dispersion will lead to incomplete summation. Nevertheless, these results seem supportive of our main conclusion: the minority of voluntary motoneuron drive originates from the CST.

Compound EPSPs from the CST are larger for motoneurons projecting more distally, especially for the intrinsic muscles of the hand (Fritz et al., 1985). It might therefore be supposed that CST inputs would be more important for hand or finger movements, compared with movements around more proximal joints such as the elbow flexion–extension task studied here. However, the limited evidence indicates that RST inputs are also stronger to intrinsic hand muscles in monkeys (Riddle et al., 2009), whereas inputs from Group Ia fibers are weaker (Clough et al., 1968). The relevant proximodistal gradient may therefore be of descending versus afferent feedback control, rather than CST versus RST. RF cells modulate their discharge during fine finger movements at least as strongly as CST cells in M1 (Soteropoulos et al., 2012). It therefore appears likely that our findings may be more generally applicable across different motoneuron pools.

In humans, the CST is more extensive than in macaques, with an estimated 2.75× more fibers, and 2.25× larger compound EPSP amplitude (Nakajima et al., 2000). It is likely, therefore, that a greater fraction of motoneuron input will come from the CST in humans than we have estimated here for macaques. However, if loud sounds enhance RF firing (which seems likely, given that humans also show a startle reflex), but suppress M1 (demonstrated in humans by Furubayashi et al., 2000), then our core finding of a dominant RF drive would remain valid, since this is the only way that StartReact could lead to reaction time shortening (Fig. 8).

Despite this conclusion, ultimately voluntary movement must arise from the cerebral cortex. The reticular formation receives powerful converging input from premotor and primary motor cortex bilaterally (Fregosi et al., 2017; Darling et al., 2018; Fisher et al., 2021); many corticoreticular fibers are collaterals of the CST (Keizer and Kuypers, 1989). Corticoreticulospinal connections could therefore be viewed as just one of many possible ways in which the motor cortex can activate motoneurons, alongside corticomotoneuronal connections, oligosynaptic pathways via segmental and propriospinal interneurons (Alstermark et al., 1999; Kinoshita et al., 2012; Takei and Seki, 2013; Witham et al., 2016), and (in monkeys, but probably not in humans) the rubrospinal system (Nathan and Smith, 1955; Mewes and Cheney, 1991; Hicks and Onodera, 2012). In addition, it is important not to view the reticulospinal system as necessarily homogeneous. Cells in PnC/PnO showed a significant short-latency facilitation after the loud sound, whereas those in Gi did not (Fig. 6*F*,*G*). This must be interpreted with caution, given that all cells from PnC/PnO were recorded in one animal; additionally, the small sample size meant that there was no statistically significant difference between the responses in each area. However, our data are at least consistent with the idea that different reticular nuclei may contribute to different functions. Previous work in rat suggested that PnC was critical to the startle reflex, whereas Gi was not (Davis et al., 1982).

### Implications for studies in humans

Since the original description of the StartReact effect by Valls-Solé et al. (1995), the method has achieved widespread use in human studies on fundamental physiology (Dean and Baker, 2017; Drummond et al., 2017; Smith et al., 2019a) and disease (Valldeoriola et al., 1998; Baker and Perez, 2017; Choudhury et al., 2019; Sangari and Perez, 2019; Rahimi and Honeycutt, 2020). Many of these publications assumed that reaction time shortening by loud sound measures the extent of RST drive. This is broadly supported by the present work (Fig. 8*D*). The StartReact effect is greater in bimanual than unimanual movements (Maslovat et al., 2020), which is compatible with the known bilateral organization of the RST (Davidson and Buford, 2006; Fisher et al., 2021). Suppression of cortical activity by TMS (the cortical silent period) delays reaction times, but the effect is smaller for movements triggered by loud sounds (Smith et al., 2019b; Teku et al., 2022); this is consistent with a reduced role of the CST. However, it is important to note that. in our simulations, when >80% of drive came from the RST, the StartReact effect did not grow further. Little reliance should be placed on this figure, which will be highly dependent on detailed model parameters, but the existence of a ceiling effect is likely to be a generally applicable result. Studies that find no change in StartReact must always consider whether they might be on the flat part of the curve relating StartReact magnitude to RST drive. It is notable, for example, that there are no differences in the size of the StartReact effect in healthy subjects performing power and precision grasps (Baker and Perez, 2017), although other evidence suggests a stronger reticulospinal contribution to power grip (Tazoe and Perez, 2017). In addition, the relationship plotted in Figure 8 depends on CST and RST cells responding to startle with early suppression and facilitation, respectively. If these responses are altered (e.g., by disease), then the validity of StartReact as a measure of RST drive may be affected.
